# Albumin nanoformulations as an innovative solution to overcome doxorubicin chemoresistance

**DOI:** 10.20517/cdr.2020.65

**Published:** 2021-03-19

**Authors:** Federica Bessone, Chiara Dianzani, Monica Argenziano, Luigi Cangemi, Rita Spagnolo, Federica Maione, Enrico Giraudo, Roberta Cavalli

**Affiliations:** ^1^Department of Drug Science and Technology, University of Turin, Turin 10125, Italy.; ^2^Laboratory of Tumor microenvironment, Candiolo Cancer Institute - FPO, IRCCS, Candiolo 10060, Italy.

**Keywords:** Albumin, nanoparticles, doxorubicin, glycol chitosan, drug resistance, ovarian and breast cancer

## Abstract

**Aim:** Resistance to chemotherapy is a major limiting factor that hamper the effectiveness of anticancer therapies. Doxorubicin is an antineoplastic agent used in the treatment of a wide range of cancers. However, it presents several limitations such as dose-dependent cardiotoxicity, lack of selectivity for tumor cells, and induced cell resistance. Nanotechnology represents a promising strategy to avoid these drawbacks. In this work, new albumin-based nanoparticles were formulated for the intracellular delivery of doxorubicin with the aim to overcome cancer drug resistance.

**Methods:** Glycol chitosan-coated and uncoated albumin nanoparticles were prepared with a tuned coacervation method. The nanoformulations were *in vitro* characterized evaluating the physicochemical parameters, morphology, and *in vitro* release kinetics. Biological assays were performed on A2780res and EMT6 cells from human ovarian carcinoma and mouse mammary cell lines resistant for doxorubicin, respectively.

**Results:** Cell viability assays showed that nanoparticles have higher cytotoxicity than the free drug. Moreover, at low concentrations, both doxorubicin-loaded nanoparticles inhibited the cell colony formation in a greater extent than drug solution. In addition, the cell uptake of the different formulations was investigated by confocal microscopy and by the HPLC determination of doxorubicin intracellular accumulation. The nanoparticles were rapidly internalized in greater extent compared to the free drug.

**Conclusion:** Based on these results, doxorubicin-loaded albumin nanoparticles might represent a novel platform to overcome the mechanism of drug resistance in cancer cell lines and improve the drug efficacy.

## Introduction

Resistance to chemotherapy is one of the main key factors that reduces the effectiveness of cancer treatment, with over 90% therapy failure rate in metastatic tumors^[1,2]^. It is well known that repeated chemotherapy administration in patients leads to the development of drug chemoresistance. Tumor cells can exploit several mechanisms to confer resistance to one or more chemotherapeutic agents. The prevalent mechanisms comprise hampered drug uptake through transporters, altered cytotoxicity against cancer cells, and increased drug efflux via active pumps, but other factors can be involved^[3]^. The resistance can involve single or multiple (multidrug resistance, MDR) drugs. In the former, the tumor can be effectively treated with alternative drugs, whereas in the latter, tumor cells become refractory to anticancer drug treatment, leading to poor clinical outcomes^[4]^. In addition, the mechanisms of anticancer drug resistance can be either intrinsic due to the innate resistance to numerous anticancer drugs or acquired when mutations occur during tumor cell proliferation. Moreover, direct or indirect anticancer drug inactivation can occur.

In this complex scenario, nanomedicine has been studied as a promising approach to overcome the several mechanisms of chemoresistance. Interestingly, nanodelivery systems, with their small sizes and peculiar physicochemical characteristics, have the potential to reverse MDR. They can affect pharmacokinetics and biodistribution profiles, favoring the accumulation in tumors either by passive or active targeting and can enhance the intracellular delivery of conventional chemotherapeutics^[5,6]^.

A number of nanocarriers, such as polymer or lipid nanoparticles (NPs), liposomes, micelles, and inorganic NPs have been proposed as a strategy to render more efficient the delivery of conventional chemotherapeutics in order to prevent the MDR onset and overcome the chemoresistance limitations associated with conventional chemotherapeutics^[7-9]^. Among organic biomaterials, albumin has been considered as one of the most useful and versatile carrier proteins in the pharmaceutical field thanks to its biocompatible, non-immunogenic, and non-toxic characteristics. Albumin-based NPs represent an ideal nanocarrier due to low cost, high availability, easy purification, and high drug-loading capability. In addition, albumin-based NPs can be easily functionalized due to the presence of charged surface groups. A large number of albumin formulations has been proposed for anticancer drug delivery^[10,11]^. Interestingly, in several recent studies doxorubicin-loaded albumin NPs have been employed as an efficient strategy to overcome the drug chemoresistance^[12-15]^.

The aim of this work was the development of new albumin nanoformulations in order to allow a more efficient intracellular delivery of doxorubicin within the tumor context and overcome cancer multidrug-resistance. Uncoated and glycolchitosan coated NPs were designed to promote cell internalization. The two new nanoformulations will be obtained with a purposely-tuned preparation method and *in vitro* characterized. The doxorubicin antiproliferative effect will be investigated in resistant cancer cell lines.

## Methods

### Materials

The substances and laboratory reagents used in this study were from Sigma-Aldrich (St Louis, MO, US), unless otherwise specified. Albumin from bovine serum (PDB ID: 3v03) was used. Bovine serum albumin (BSA) is a single polypeptide chain consisting of about 583 amino acid residues (sequence: DTHKSEIAHRFKDLGEEHFKGLVLIAFSQYLQQCPFDEHVKLVNELTEFAKTCVADESHAGCEKSLHTLFGDELCKVASLRETYGDMADCCEKQEPERNECFLSHKDDSPDLPKLKPDPNTLCDEFKADEKKFWGKYLYEIARRHPYFYAPELLYYANKYNGVFQECCQAEDKGACLLPKIETMREKVLTSSARQRLRCASIQKFGERALKAWSVARLSQKFPKAEFVEVTKLVTDLTKVHKECCHGDLLECADDRADLAKYICDNQDTISSKLKECCDKPLLEKSHCIAEVEKDAIPENLPPLTADFAEDKDVCKNYQEAKDAFLGSFLYEYSRRHPEYAVSVLLRLAKEYEATLEECCAKDDPHACYSTVFDKLKHLVDEPQNLIKQNCDQFEKLGEYGFQNALIVRYTRKVPQVSTPTLVEVSRSLGKVGTRCCTKPESERMPCTEDYLSLILNRLCVLHEKTPVSEKVTKCCTESLVNRRPCFSALTPDETYVPKAFDEKLFTFHADICTLPDTEKQIKKQTALVELLKHKPKATEEQLKTVMENFVAFVDKCCAADDKEACFAVEGPKLVVSTQTALA) without carbohydrates. At pH 5-7 it contains 17 intrachain disulfide bridges and 1 sulfhydryl group. Ultrapure water was obtained using a 1-800 Millipore system (Molsheim, France). The tubular semi-permeable cellulose membrane was from Carl Roth (Karlsruhe, Germany). Cell culture reagents were purchased from Gibco/Invitrogen (Life Technologies, Paisley, UK) except where otherwise indicated. All reagents were of analytical grade.

### Preparation of albumin nanoparticles

In order to obtain stable and reproducible albumin NPs, a preformulation study was carried out. Different BSA concentrations were tested with different amounts of the nonionic surfactant Polysorbate 80 to reach the optimal conditions to load doxorubicin hydrochloride (DOX).

#### Albumin nanoparticles

Once optimized the process, blank BSA NPs were prepared based on a tuned coacervation method. Briefly, 25 mg of BSA were dissolved in 1 mL of 50 mmol/L TRIS buffer at pH 7.4 containing Polysorbate 80 (2% w/v) as surfactant. Then, ethanol was added dropwise under sonication in a 220-230 V Bransonic® 3510 Ultrasonic Cleaner (Emerson; Saint Louis, MO, US) at 60 °C for 1 h. When the nanosuspension was obtained, the ethanol was evaporated under nitrogen flux. Moreover, glycol chitosan-coated nanoparticles (blank GC-NPs) were obtained by adding dropwise under constant stirring an aqueous solution of glycol chitosan (2.7% w/v) to the preformed blank BSA-NPs.

#### Doxorubicin-loaded albumin nanoparticles

Albumin NPs loaded with doxorubicin were obtained by a purposely tuned coacervation method. Briefly, 25 mg of BSA were dissolved in 1 mL of 50 mmol/L TRIS buffer at pH 7.4 containing Polysorbate 80 (2% w/v). DOX was dissolved in the BSA solution with a concentration of 3 mg/mL Then, ethanol was added dropwise under sonication in a 220-230 V Bransonic® 3510 Ultrasonic Cleaner (Emerson; Saint Louis, MO, US) at 60 °C for 1 h. When the nanosuspension was obtained, the ethanol was evaporated under nitrogen flux. In addition, glycol chitosan-coated nanoparticles (GC DOX-NPs) were obtained by adding dropwise under constant stirring an aqueous of glycol chitosan (2.7% w/v) to the preformed DOX-loaded nanoparticles (DOX-NPs).

### Physicochemical characterization of albumin nanoparticles

The average diameter, dispersity and zeta potential were measured by photocorrelation spectroscopy using a particle size analyser at a scattering angle of 90° and a temperature of 25 °C. NP suspensions were diluted in deionized filtered water before measurement. For zeta potential determination, already diluted NP formulations were placed in the electrophoretic cell, where an electric field of approximately 15 V/cm was applied. The morphology of formulations was evaluated by Transmission Electron Microscopy (TEM), using a Philips CM10 (Eindhoven, NL) instrument. Plain NPs and DOX-NP nanosuspensions were sprayed on Formvar-coated copper grid and air-dried before observation.

Thermal analysis was carried out using a DSC/7 differential scanning calorimeter (Perkin-Elmer, Branford, CT, US) equipped with a TAC 7/DX instrument controller and the Pyris program. A heating rate of 10 °C per min was used in the 30-230 °C temperature range. Standard aluminium sample pans for solids (Perkin-Elmer) were used and about 20 mg of the freeze-dried NPs weighed. Doxorubicin and BSA-NPs were analyzed for comparison purposes. Attenuated Total Reflection (ATR)-Fourier transformed infrared (FTIR) spectra of the DOX-NPs, GC DOX-NPs, free DOX, and blank BSA-NPs were obtained using a Perkin Elmer Spectrum 100 FT-IR in the region 4000^-1^-650^-1^.

The stability of the nanoformulations stored at 4 °C was evaluated overtime, checking the physicochemical parameters and the doxorubicin concentration of the NPs.

### HPLC quantitative doxorubicin determination

HPLC system consisted of a pump (LC-9A PUMP C, Shimadzu, Japan) equipped with a fluorescence detector (Chrompack, Japan) and analyses were performed using an Agilent TC C18 column (250 mm × 4.6 mm, 5 µm). A degassed solution containing 65% v/v phosphate buffer (pH = 1.4), 25% v/v acetonitrile, and 10% distilled water was used as mobile phase. The flow rate was 1 mL/min and the fluorimetric detector (Shimadzu) was set at λ_exc_ = 480 nm and λ_em_ = 560 nm. To calculate the drug concentration, a linear calibration curve was set up with a concentration range of 0.25-2.5 μg/mL with a regression coefficient of 0.999.

### Encapsulation efficiency and loading capacity

The encapsulation efficiency of DOX-NPs and GC DOX-NPs was determined with a centrifugal filter system. A part of the two nanoformulations was placed in a centrifugal filter device (Amicon® Ultra-0.5) and centrifuged for 15 min at 15,000 rpm with a Beckman Coulter 64R Centrifuge. Then, the filtered solution containing free DOX was analyzed at the HPLC as previously described. The encapsulation efficiency of DOX was quantified by Equation 1.

Encapsulation Efficiency (EE):

**Figure:**
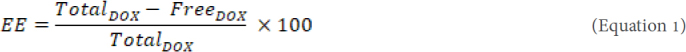


The loading capacity was determined on freeze-dried NP samples. Briefly, a weighted amount of freeze-dried DOX-NPs and GC DOX-NPs was suspended in 10 mL of filtered water. After sonication and centrifugation, the supernatant was diluted with mobile phase and analyzed by HPLC. The loading capacity was calculated by Equation 2.

Loading Capacity (LC):

**Figure:**
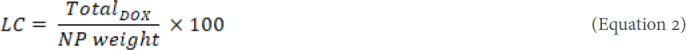


### *In vitro* doxorubicin release studies

The *in vitro* release of DOX from the NPs was carried out with a multi-compartment rotating cell. DOX-NPs, GC DOX-NPs, and DOX solution as control were used as donors against a receiving phase consisting of phosphate buffered saline pH 7.4. The chambers were separated with a semi-permeable cellulose membrane (cut-off 14kDa). Samples were drawn at fixed time intervals and the same volume was replaced with fresh medium. Then, DOX concentration was determined using HPLC, as previously described.

#### Mechanism of drug release

*In vitro* release profiles of doxorubicin from DOX-loaded albumin-nanoformulations were analyzed to assess the mechanism of drug release using four kinetic models: zero-order kinetic model, first-order kinetic model, simplified Higuchi model, and Korsmeyer-Peppas model^[16]^. Briefly, for each model, a graph was constructed using Microsoft Excel from which the rate constant and correlation values were obtained by applying a linear regression fit. The zero-order kinetic model was obtained by plotting cumulative percent drug release versus time. The first-order kinetic model was analyzed by plotting log cumulative percent of drug remaining versus time. The Higuchi model was evaluated by plotting cumulative percent drug release versus square root of time, while the Korsmeyer-Peppas model was analyzed by plotting log cumulative percent drug release versus log time.

### Cells

To evaluate the contribution of doxorubicin-loaded albumin-based NPs, two different cell lines resistant to doxorubicin were employed. A2780res human ovarian carcinoma cells, and EMT6/AR10 murine cancer mammary cells were incubated at 37 °C with 5% CO_2_ atmosphere.

EMT6/AR10 cells were cultured in MEM (EBSS) medium, whereas A2780res cells were cultured in RPMI. All the media were supplemented with 10% v/v Fetal Bovine Serum (FBS), 2 mmol/L Glutamine, and antibiotics. To maintain the resistance, A2780 ADR and MCF7 ADR were cultured in media containing 100 nmol/L doxorubicin HCl twice a week.

### Cell viability assay

The cytotoxic effect of DOX-NPs and GC DOX-NPs, and of their blank counterparts was evaluated. The EMT6/AR10 and A2780res cells were seeded in a 96-well plate (3 × 10^3^ cells/well) and incubated with the albumin-based nanoformulations at different DOX concentration. After 24 h and 48 h of incubation, viable cells were evaluated by 3-(4,5-Dimethyl-2-thiazolyl)-2,5-diphenyl-2H-tetrazolium bromide (MTT) inner salt reagent at 570 nm. The readouts from treated cells were expressed as percentage versus control measured on untreated cells.

### Colony-forming assay

A2780res cells were seeded into 6-well plates and treated with different concentrations of DOX-NPs, GC DOX-NPs, and DOX solution as control. The medium was changed respectively after 3 h and 20 h and cells were cultured for an additional 10 days. After that, the cells were fixed and stained with a solution of 80% v/v crystal violet and 20% v/v of ethanol. To induce a complete dissolution of the crystal violet, a solution of 30% v/v acetic acid was added and the absorbance was detected at 595 nm.

### Study of doxorubicin-loaded BSA-NP internalization in A2780res cells

A2780res cells were seeded onto Corning® cover glasses (Sigma-Aldrich) in a 24-well plate (4 × 10^4^ cells/ well) and incubated overnight at 37 °C in a 5% CO_2_ atmosphere. Then, the cells were incubated with DOX-NPs, GC DOX-NPs, and DOX solution at a final concentration of 5 µg/mL for 1 h. After that, the cells were washed with PBS, fixed with 4% Paraformaldehyde (PAF) for 15 min at room temperature, and stained with 4’,6-diamidine-2-phenylindole (DAPI). Finally, coverslips were mounted. The samples were analyzed with a TCS SP2 AOBS confocal microscope (Leica Leica, Wetzlar, Germany) equipped with 63X/1.40 HCX Plan-Apochromat oil-immersion objective.

### Evaluation of doxorubicin intracellular concentration

The cellular uptake of DOX-loaded albumin NPs was evaluated measuring the intracellular doxorubicin concentration by HPLC analysis. The A2780res cells were seeded in a 96-well plate (3 × 10^3^ cells/well) and incubated with the albumin nanoformulations at different DOX concentration (1, 5, and 10 µmol/L) for 2 h and 4 h at 37 °C. Following the incubation, the cells were washed three times with phosphate buffered saline (PBS 1X) and lysed with a saturated solution of ammonium sulphate. After centrifugation (10 min at 13,000 rpm at 4 °C) cell lysates were frozen and stored at -80 °C. Immediately prior to the analysis, cell lysates were thawed and centrifuged (10 min at 13,000 rpm at 10 °C). The supernatants were analyzed by HPLC after suitable dilution with the mobile phase, as described above, to determine the amount of doxorubicin inside the cells. The experiment was performed in triplicate.

### Confocal analysis

Cells were grown on glass coverslips for 24 h. Then, A2780res were incubated for 1 h with DOX-NPs, GC DOX-NPs, and DOX solution. After rinse, cells were fixed in 4% formaldehyde for 10 min at room temperature and DAPI was used to counterstain the nuclei. Fluorescent images were acquired using a Leica TCS SP5 AOBS microscope (Leica Microsystems).

### Statistical analysis

Data are presented as mean ± standard deviation (SD). Statistical analyses were performed with GraphPad Prism program (version 6; GraphPad Software). Two-tailed Student’s *t*-tests were performed to analyze the statistical significance between two groups.

## Results

**Figure 1:**
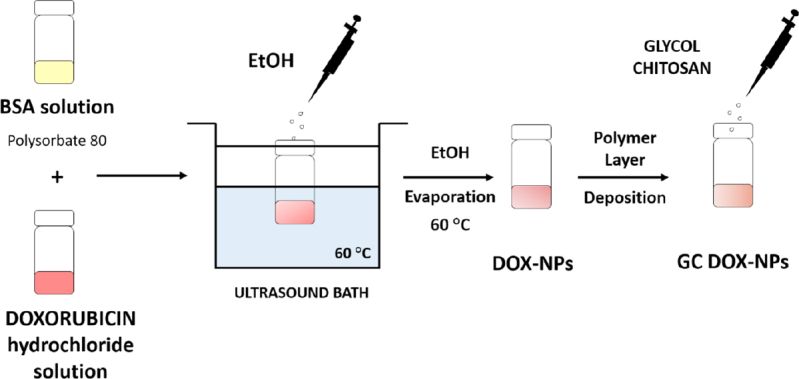
Representative scheme of the tuned preparation method of uncoated (DOX-NPs) and glycol chitosan-coated (GC DOX-NPs) DOX-loaded albumin nanoparticles. DOX: doxorubicin hydrochloride; GC: glycol chitosan-coated; NPs: nanoparticles

Albumin NPs were purposely designed and developed for the cellular delivery of doxorubicin. A preparation process was tuned to obtain stable and reproducible formulations. The schematic representation of the preparation method is reported in Figure 1. Two types of doxorubicin-loaded albumin NPs were prepared, uncoated (DOX-NPs) and glycol chitosan-coated (GC DOX-NPs).

### Physicochemical characterization of nanoparticles

The physicochemical parameters of BSA-NPs are reported in Table 1.

**Table 1:** Physicochemical characterization of albumin nanoparticles

BSA Nanoparticles	Average diameter (nm) ± SD	Đ	Z-potential (mV) ± SD	EE (%)	LC (%)
Blank BSA-NPs	362.1 ± 7.6	0.21	- 27.3 ± 2.3	-	-
DOX-NPs	380.3 ± 9.5	0.26	- 28.9 ± 4.1	90.2 ± 1.3	6.4 ± 0.8
Blank GC BSA-NPs	365.3 ± 12.8	0.23	7.6 ± 0.9	-	-
GC DOX-NPs	383.6 ± 10.2	0.21	8.0 ± 0.5	85.2 ± 1.2	4.3 ± 0.4

Results are shown as means ± standard deviation from three different preparations (*n* = 10). Đ: dispersity; EE: encapsulation efficiency; LC: loading capacity; BSA: bovine serum albumin; NPs: nanoparticles; DOX: doxorubicin hydrochloride; GC: glycol chitosan-coated

All the formulations presented an average diameter between 360 and 385 nm. The uncoated formulations presented a negative surface charge due to the contribution of the carboxyl groups of albumin. On the other hand, the presence of the coating of glycol chitosan did not modify the sizes of the NPs but affected their surface charges. Indeed, when coated with glycol chitosan, the zeta potential of the nanoformulations turned to positive because of the amine groups of the polymer. The increased zeta potential confirmed the interaction between the albumin and the polymer. All the formulations presented a physiological pH in the range 6.8-7.4.

**Figure 2:**
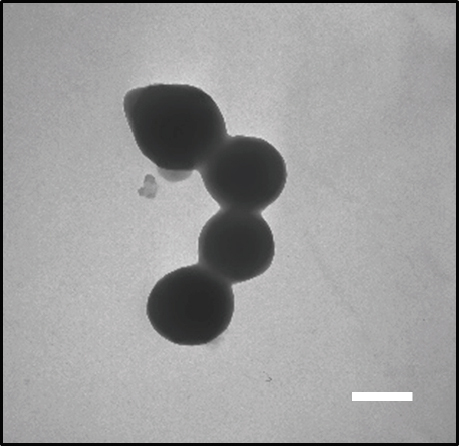
Transmission electron microscopy image of GC DOX-NPs (magnification 28,500 ×). Results are presented as a representative image from three different preparations. Scale bar, 300 nm. DOX: doxorubicin hydrochloride; GC: glycol chitosan-coated; NPs: nanoparticles

The spherical morphology of albumin nanoformulations and their nanometric sizes were confirmed by TEM analysis. All the NPs presented a reproducible spherical shape. As an example, the morphological appearance of GC DOX-NPs is presented in Figure 2.

**Figure 3:**
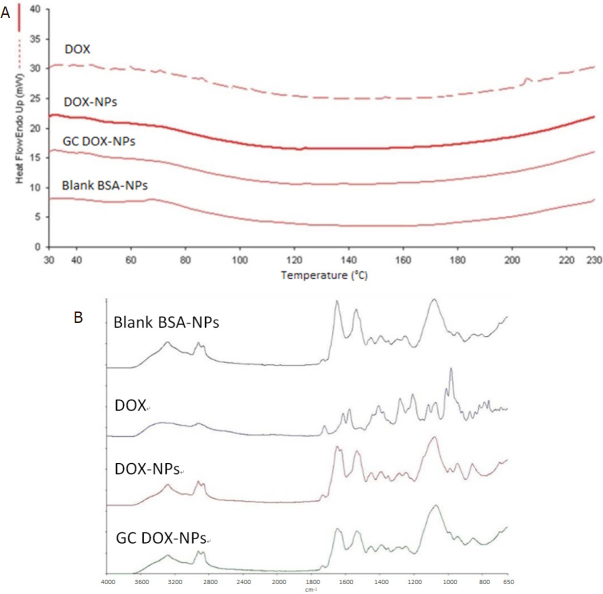
DSC thermograms (A) and ATR-FTIR spectra (B) of DOX solution, DOX-NPs, GC DOX-NPs, and blank BSA-NPs confirmed the interaction between the drug and the nanoparticles. DSC: Differential Scanning Calorimetry; BSA: bovine serum albumin; DOX: doxorubicin hydrochloride; GC: glycol chitosan-coated; DOX-NPs: DOX-loaded nanoparticles

The interaction of the drug with albumin was investigated by Differential Scanning Calorimetry (DSC) and ATR-FTIR analyses. DSC thermograms and ATR-FTIR spectra of free DOX, DOX-NPs, GC DOX-NPs, and blank BSA-NPs were shown in Figure 3A and B, respectively.

The disappearance of the endothermic peak related to doxorubicin melting point (at about 220 °C) in the thermogram of DOX-NPs confirmed the interaction between doxorubicin and albumin. Moreover, FTIR spectra confirmed the drug incorporation in the albumin NPs.

Both albumin NPs were stable up to 1 year, stored at 4 °C. Indeed, no changes in the physicochemical characteristics (size and surface charge) and no aggregation phenomena were observed. The doxorubicin content in the albumin NPs did not decreased overtime.

### *In vitro* release studies

**Figure 4:**
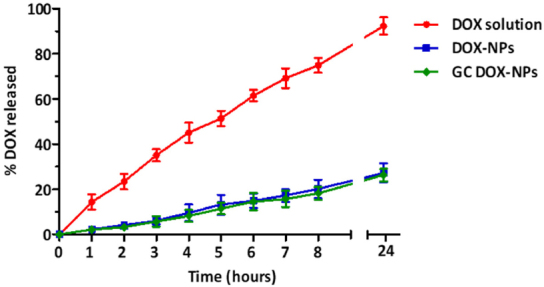
*In vitro* DOX release from DOX-NPs and GC DOX-NPs with DOX solution as control, up to 24 h. Each point represents the mean ± SD of three different formulations. DOX: doxorubicin hydrochloride; GC: glycol chitosan-coated; DOX-NPs: DOX-loaded nanoparticles

The *in vitro* release kinetics profile of doxorubicin from DOX-NP and GC DOX-NP formulations is showed in Figure 4.

For both formulations, the release of the drug was slow and prolonged over time, compared to free solution diffusion. After 24 h, only 30% of the encapsulated drug was released from the DOX-NPs and GC DOX-NPs. On the other hand, the solution of DOX hydrochloride passed completely into the receiving phase. No initial burst effect was observed indicating that the drug is not absorbed on the nanoparticle surface but incorporated. The coating with glycol chitosan did not affect the doxorubicin release profile of GC DOX-NPs.

**Figure 5:**
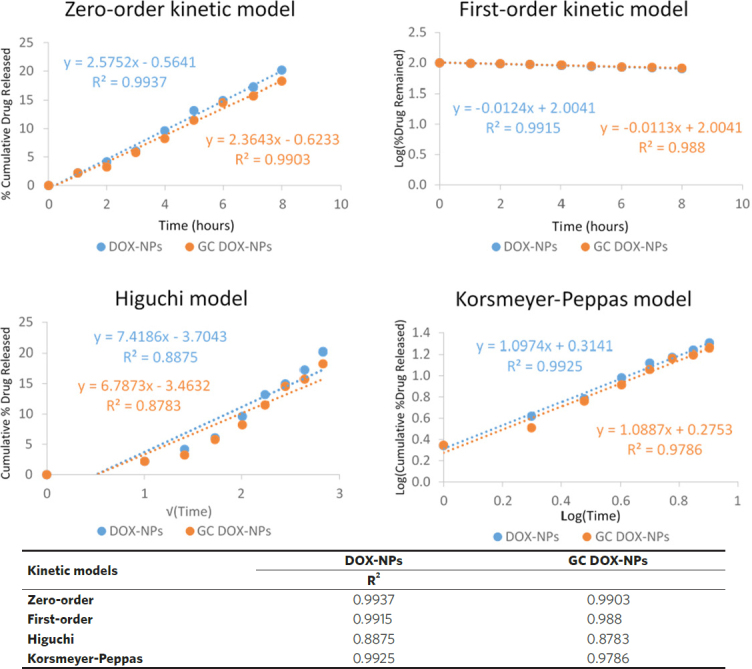
Doxorubicin release from DOX-NPs and GC DOX-NPs (points, representing average values) fitted to kinetic models (lines). The *in vitro* release kinetics was fitted to each kinetic model by plotting: cumulative percent drug release *vs*. time for zero-order kinetic model, log of perscent drug remaining *vs*. time for first-order kinetic model, cumulative percent drug release versus square root of time for simplified Higuchi model, and log cumulative percent drug release versus log time for Korsmeyer-Peppas model. For DOX-NPs, the zero-order kinetic, the first-order kinetic and the Korsmeyer-Peppas models showed a high correlation with R^2^ > 0.99. For GC DOX-NPs, the zero-order kinetic showed R^2^ > 0.99. The *R*^2^ values are reported in the table. DOX: doxorubicin hydrochloride; GC: glycol chitosan-coated; DOX-NPs: DOX-loaded nanoparticles

The sustained release may be primarily due to the slow diffusion of doxorubicin across the albumin matrix of NPs. This could represent a favorable condition to minimize the systemic adverse side effects of doxorubicin and to enhance the efficiency of NPs at the target site. In addition, the mechanism of drug release was also analyzed using four mathematical kinetics models corresponding to: zero-order kinetics, first-order kinetics, simplified Higuchi, and Korsmeyer-Peppas models. For each kinetics model, a graph was constructed in Microsoft Excel from which the rate constant and correlation values were obtained by applying a linear regression fit [Figure 5]. The *in vitro* release experimental results best fitted with the simplified zero-order kinetic for both DOX-NPs and GC DOX-NPs, indicating that the doxorubicin is released from the NPs by passive diffusion through the albumin matrix. Indeed, zero-order kinetics fits with nanostructures, the release of which is not due to the erosion of the matrix. In fact, albumin NPs provide constant and slow release kinetics of the encapsulated drugs.

### Doxorubicin-loaded albumin nanoparticles impaired A2780res and EMT6/AR10 viability and proliferation

**Figure 6:**
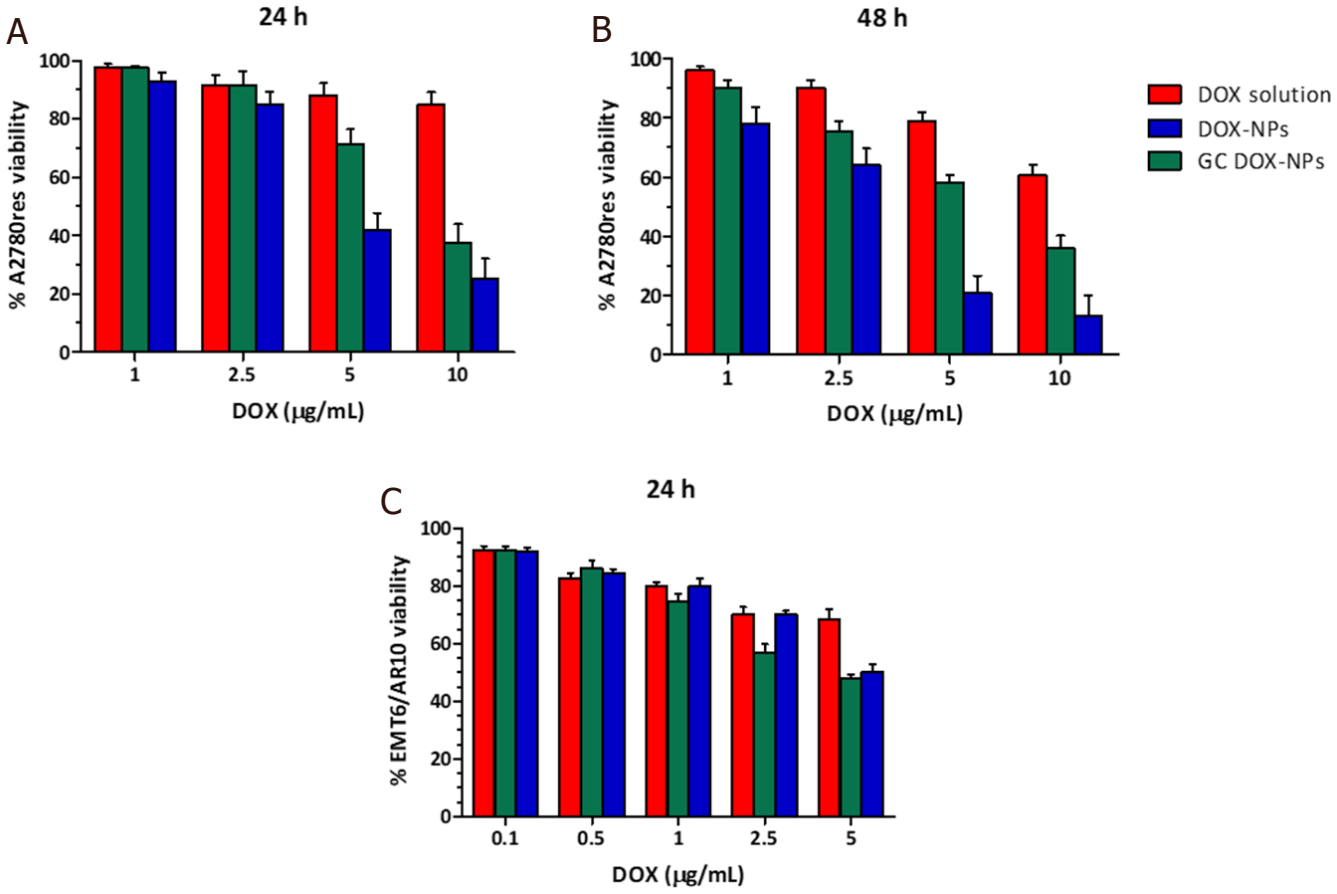
DOX solution, DOX-NPs, and GC DOX-NPs were incubated with A2780res and EMT6/AR10 cells for 24 h (A, C) and 48 h (B) and the cell viability was evaluated. Each bar represents the mean ± SD of five different experiments (*n* = 3). DOX: doxorubicin hydrochloride; GC: glycol chitosan-coated; DOX-NPs: DOX-loaded nanoparticles

The cytotoxicity of DOX solution, DOX-NPs, GC DOX-NPs, and their blank counterparts was evaluated *in vitro* on A2780res and EMT6/AR10 cell lines, with a Doxorubicin resistant profile. After 24 h and 48 h of incubation, DOX-NPs and GC DOX-NPs inhibited A2780res and EMT6/AR10 viability to a greater extent than free DOX [Figure 6]. After 24 h, A2780res presented 20% of cell viability when incubated with DOX-NPs 10 μg/mL, while EMT6/AR10 cells just 50% cell viability. After 48 h, the viability was even more inhibited. On the other hand, GC DOX-NPs showed to be slightly less effective, giving a cell viability of 40% after 24 h and 65% after 48 h for A2780res. Instead, in the EMT6/AR10 the cytotoxicity was around 50%. Besides, the DOX solution presented cytotoxicity of less than 40% after 48 h of incubation. Taken together, the NPs resulted to be more effective *in vitro* than free DOX solution because they acted as “trojan horses,” reducing the contribution of the P-gp in pumping out the drug from the cells. The NPs remain inside the cells, where they release the drug for a prolonged time.

Moreover, IC_50_ values of free DOX, DOX-NP, and GC DOX-NP in A2780res were calculated [Table 2].

**Table 2:** Determination IC_50_ values of free DOX, DOX-NPs, and GC DOX-NPs in A2780res cells after 24 h and 48 h of incubation

Cell line	Time of incubation	DOX IC_50_ (μmol/L)	DOX-NPs IC_50_ (μmol/L)	GC DOX-NPs IC_50_ (μmol/L)
A2780res	24 h	1.2 × 10^-1^ ± 0.60	5.6 × 10^-3^ ± 0.50*	8.2 × 10^-3^ ± 0.50*
	48 h	2.7 × 10^-2^ ± 0.60	9.8 × 10^-5^ ± 1.60*	3.7 × 10^-3^ ± 0.90

Results are mean ± SD for three different experiments. *P < 0.05. NPs: nanoparticles; DOX: doxorubicin hydrochloride; GC: glycol chitosan-coated

**Figure 7:**
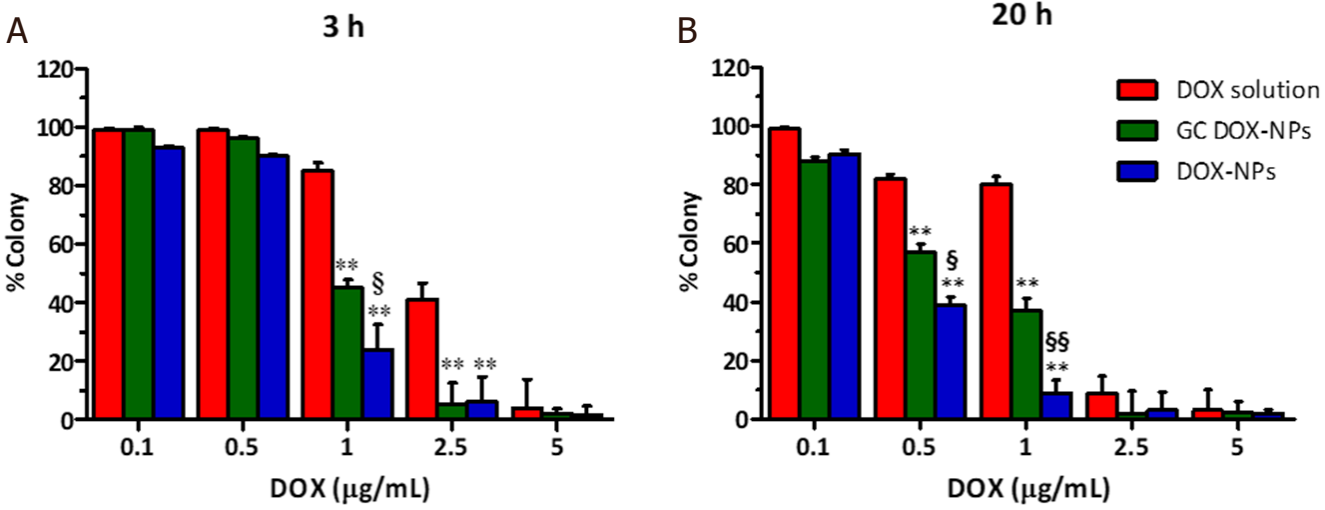
Doxorubicin-loaded albumin nanoparticles impaired A2780res proliferation. Clonogenic assay was performed at 3 h (A) and 20 h (B) of incubation with increasing concentration of DOX-NPs, GC DOX-NPs, and DOX solution. **P < 0.01 *vs*. DOX solution; ^§^*P* < 0.05, ^§§^*P* < 0.01 *vs*. GC DOX-NPs. DOX: doxorubicin hydrochloride; GC: glycol chitosan-coated; DOX-NPs: DOX-loaded nanoparticles

The anti-proliferative effect of the albumin doxorubicin-loaded NPs was tested *in vitro*. The cells were incubated for 3 h and 20 h with the different formulations, and then cultured for additionally 10 days. After that time, the colony was evaluated. As shown in Figure 7, DOX-NPs and GC DOX-NPs significantly hampered the proliferation of A2780res cells after just 3 h of incubation at a concentration of 1 μg/mL. After 20 h of incubation, instead, the nanoformulations was found to be effective starting from 0.5 μg/mL of the drug.

### Doxorubicin-loaded albumin nanoparticle internalization in A2780res cells

**Figure 8:**
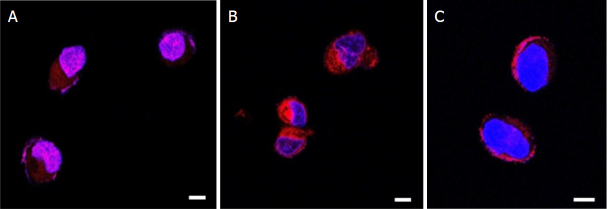
DOX-NPs were internalized in A2780res and entered in cell nuclei. A2780res cells were incubated with DOX-NP (A), GC DOX-NP (B), and DOX solution (C) for 1 h. Laser scanning confocal microscope images at 63X. Scale bar, 4 μm. DOX: doxorubicin hydrochloride; GC: glycol chitosan-coated; DOX-NPs: DOX-loaded nanoparticles

In order to study the ability of our formulations to facilitate the doxorubicin entry within the cells, we performed a confocal analysis on A2780res cells treated with DOX-NPs, GC DOX-NPs, and DOX solution. Confocal analysis was performed by exploiting DOX fluorescence, whereas DAPI was used to visualize the nuclei. The images reported in Figure 8 showed that the NPs were more avidly and rapidly internalized by the cells compared with DOX solution [Figure 8A and B], which localized at the edges of the cell membrane, probably at the efflux pump level [Figure 8C]. Notably, more drug seems to accumulate in the cytoplasm in A278res cells treated with GC DOX-NP compared with uncoated NPs. These results clearly confirm that albumin nanoparticle facilitate drug internalization also in DOX-resistant cell lines.

**Figure 9:**
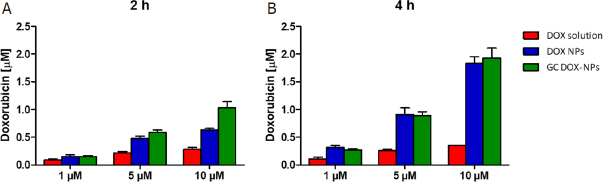
Intracellular doxorubicin concentration (μmol/L) in A2780res cells. A2780res cells were incubated with DOX-NP, GC DOX-NP, and DOX solution at different DOX concentration (1, 5, 10 μmol/L) for 2 h (A) and 4 h (B). Then, the cells were lysed, and cell lysates were analyzed by HPLC. The results represent the mean ± SD. DOX: doxorubicin hydrochloride; GC: glycol chitosan-coated; DOX-NPs: DOX-loaded nanoparticles

Moreover, the cellular uptake of DOX-loaded NPs was investigated to determine the intracellular DOX concentration in A2780res cells by HPLC analysis [Figure 9].

A higher intracellular accumulation of the drug in cells treated with both DOX-NPs was observed in comparison with free drug-treated cells. In particular, the highest intracellular DOX concentrations were found after incubation with GC DOX-NPs. These results are in agreement with the confocal analyses.

## Discussion

Multidrug resistance (MDR) represents a big barrier for the effectiveness of anticancer drugs, and it can be attributed to the over-expression of ATP-binding cassette (ABC) transporter, which causes drug efflux.

The nanoparticle technology can play an important role in counteracting the development of drug resistance in different cancer diseases^[17]^. Indeed, nanomedicine can contribute to escape the mechanism of efflux pumps (i.e., P-glycoproteins and multidrug-resistance proteins), which are overexpressed in MDR cancer cells. The NPs avoid being pumped out from the cells, thus increasing the amount of the carried chemotherapeutic and its efficacy^[18]^.

Interestingly, Salomon and Ehrhardt^[19]^ showed that the physicochemical characteristics of NPs can affect the membrane transporter function.

It is worth noting that doxorubicin-based chemotherapy can fail either for inherent or acquired resistance^[20]^. DOX can confer drug resistance in a mechanism that involves an altered or increased expression of ABC transporters. Through this mechanism, cancer cells can easily efflux DOX through up-regulating efflux transporters, with a consequent decrease in intracellular drug concentration and effectiveness. Thus, to enhance the chemotherapeutic outcome, high drug doses would be necessary causing severe systemic toxicity^[21]^. The nanotechnological approach may be a promising solution. In fact, a liposomal doxorubicin nanoformulation (Doxil®) is on the market but much research is still focused on the design of nanocarriers for the delivery of doxorubicin to tumor cells^[22-27]^.

Interestingly, the co-delivery of doxorubicin combined with modulators of drug resistance within one nanocarrier has shown to be effective in overcoming MDR mediated efflux^[28,29]^. In this study, BSA-based NPs were formulated for doxorubicin intracellular delivery aimed at overcoming the drug resistance.

Albumin was selected for the NP manufacturing because it is a component of the human blood, a notable carrier protein with multiple cellular receptors and binding sites, and a protein capable of transporting different endogenous and exogenous molecules. Albumin from bovine serum, cost effective compared to human albumin, was used in this study which consisted of a formulation design of new albumin NPs. However, preliminary experiments confirmed that the purposely tuned preparation method described in this paper can also be adopted for the preparation of human albumin NPs.

Worthy of note is the FDA approval of Abraxane® in 2005, which is a paclitaxel albumin-bound nanoparticle formulation for the treatment of metastatic breast cancer, showing a significantly longer time prior to tumor progression with respect to the free drug. However, in the literature a few studies demonstrated that nanoparticle albumin-bound (nab)-paclitaxel can induce drug resistance by up-regulating P-gp^[30,31]^.

In this contest, albumin nanosystems have been previously proposed for the delivery of doxorubicin to overcome multidrug resistance^[13-15]^. In the literature different techniques for the albumin NP fabrication were explored such as desolvation, chemical crosslinking, emulsification, thermal gelation, spray drying, and self-assembly^[32]^. Here, a coacervation method was purposely tuned to obtain stable and reproducible albumin NPs. The procedure is cost-effective and up-scaled manufacturing can be achieved.

Two types of albumin-based formulations were developed, such as uncoated and glycolchitosan coated albumin-NPs. The design criteria took into account that size, shape, and surface charge are the key parameters to affect NP biodistribution and cell internalization. The glycolchitosan coating was added to reduce the negative surface charge of albumin NPs in order to favor their interaction with the cellular membrane. In fact, positively charged NPs are taken up more easily than negatively charged ones. Nanocarriers with positive zeta potential bind more strongly to the cell membrane, facilitating the NP internalization. Glycolchitosan coating provides a positive surface charge to albumin nanoformulations. The strong zeta potential variation of NPs confirmed the interaction between the polymer chains and albumin.

Interestingly, the modification of NP surface with hydrophilic polysaccharides is an alternative to PEGilation to modify pharmacokinetics parameters of NPs^[33]^.

The two types of albumin NPs were able to load doxorubicin in a good extent, reaching a loading capacity of 4.3% and 6.4 % for GC DOX-NPs and DOX-NPs, respectively. The encapsulation efficiency was above the 85% for both nanoformulations. These percentages might be sufficient for *in vivo* efficacy in animal models. Indeed, a peculiar characteristic of nanoparticulate systems is to enable a dose reduction, due the different pharmacokinetics profile compared to the free drug, as demonstrated also for Doxil®^[34]^.

The *in vitro* release study showed that the drug is released in a constant and sustained manner over time.

In addition, the coating did not affect the doxorubicin release profile from NPs. This result suggests that the polymer covers the albumin NP as a thin layer on the surface.

Cell viability assay showed that albumin NPs were able to enhance doxorubicin antiproliferative effect in resistant cancer cells in comparison to doxorubicin solution. Notably, a significant decrease of IC50 values was observed for coated and uncoated albumin NPs. These results might suggest that albumin NPs are able to be internalized and might play a role in decreasing P-gp efflux pump activity. This hypothesized behavior of albumin NPs can be a promising advantage as not all nanoformulations are able to overcome efflux-mediated resistance. Recently, Pieper *et al*.^[35]^ evaluated the anticancer activity of doxorubicin incorporated in PLA- and PLGA-based NPs on P-gp expressing cancer cells showing that the investigated NPs did not bypass transporter-mediated drug efflux^[35]^. Confocal microscopy analysis underlined that our albumin NPs are readily internalized in tumor cells with nuclear localization. On the contrary, doxorubicin in solution is mainly localized at cell surfaces. Through confocal analysis, we clearly demonstrated that both the formulations were more efficient for drug internalization compared with free doxorubicin. The internalization capability of DOX-NPs and GC DOX-NPs was confirmed by the cell accumulation study. The intracellular doxorubicin concentration after the incubation with albumin NPs was greater than with doxorubicin solution. For example, in cells treated with 10 µmol/L dose an increase in DOX intracellular concentration of 5.5 folds compared to free DOX was reached. The highest DOX accumulation was observed for CG DOX-NPs, confirming the role of glycol chitosan coating in promoting cell internalization. Together our findings indicate that these albumin NPs might be effective to overcome MDR.

In conclusion, it is worth noting that resistance to chemotherapy limits the effectiveness of anticancer drug treatment. Overcoming this limitation can the change the survival of cancer. In this work, a reproducible coacervation method to obtain albumin-NPs with narrow size distribution was fine-tuned. Notably, glycol chitosan-coated and uncoated albumin-based doxorubicin-loaded NPs were obtained. These albumin NPs significantly inhibited the *in vitro* viability and proliferation of cell lines resistant to doxorubicin, thus demonstrating the importance of the nanoformulation to counteract drug resistance. Taken together, these *in vitro* results showed that GC DOX-NPs and DOX-NPs may represent a novel platform to affect the mechanism of drug resistance in cancer cell lines, improving the efficacy of the chemotherapy.

## Declarations

### Copyright

© The Author(s) 2021.
